# Combining single-cell RNA sequencing and network pharmacology to explore the target of cangfu daotan decoction in the treatment of obese polycystic ovary syndrome from an immune perspective

**DOI:** 10.3389/fphar.2024.1451300

**Published:** 2024-10-30

**Authors:** Danqi Liu, Chaofeng Wei, Lu Guan, Wenhan Ju, Shan Xiang, Fang Lian

**Affiliations:** ^1^ The First Clinical Medicine School, Shandong University of Traditional Chinese Medicine, Jinan, Shandong, China; ^2^ Department of Biomedical and Molecular Sciences, Queen’s University, Kingston, ON, Canada; ^3^ Integrative Medicine Research Centre of Reproduction and Heredity, Affiliated Hospital, Shandong University of Traditional Chinese Medicine, Jinan, Shandong, China

**Keywords:** polycystic ovary syndrome, immune abnormalities, obesity, single-cell RNA sequencing, network pharmacology, cangfu daotan decoction

## Abstract

**Background:**

Polycystic ovary syndrome (PCOS) is a heterogeneous gynecological endocrine disorder linked to immunity. Cangfu Daotan Decoction (CFDT), a classic Chinese medicine prescription, is particularly effective in treating PCOS, specifically in patients with obesity; however, its specific mechanism remains unclear.

**Methods:**

Part 1: Peripheral blood mononuclear cells were collected on egg retrieval day from obese and normal-weight patients with PCOS and healthy women undergoing *in vitro* fertilization (IVF)-embryo transfer. Next, scRNA-seq was performed to screen the key genes of bese patients with PCOS. Part 2: Active ingredients of CFDT and obesity-related PCOS targets were identified based on public databases, and the binding ability between the active ingredients and targets was analyzed. Part 3: This part was a monocentric, randomized controlled trial. The obese women with PCOS were randomized to CFDT (6 packets/day) or placebo, and the healthy women were included in the blank control group (43 cases per group). The clinical manifestations and laboratory outcomes among the three groups were compared.

**Results:**

Based on the scRNA-seq data from Part 1, CYLD, ARPC3, CXCR4, RORA, JUN, FGL2, ZEB2, GNLY, FTL, SMAD3, IL7R, KIR2DL1, CTSD, BTG2, CCL5, HLA, RETN, CTSZ, and NCF2 were potential key genes associated with obese PCOS were identified. The proportions of T, B, and natural killer cells were higher in patients with PCOS compared to healthy women, with even higher proportions observed in obese patients with PCOS. Gene ontology and the Kyoto encyclopedia of genes and genomes analysis depicted that the differentially expressed genes were related to immune regulation pathways. Network pharmacology analysis identified that the key active components in CFDT were quercetin, carvacrol, β-sitosterol, cholesterol, and nobiletin, and TP53, AKT1, STAT3, JUN, SRC, etc. were the core targets. The core targets and their enrichment pathways overlapped with those in Part 1. Clinical trials in Part 3 found that CFDT reduced the dosage of gonadotropins use in patients with PCOS, increased the number of high-quality embryos, and improved the ongoing pregnancy rate.

**Conclusion:**

CFDT can improve the immune microenvironment of patients to some extent, reduce their economic burden, and enhance IVF outcomes. The improvement in the immune microenvironment in obese patients with PCOS may be linked to targets such as JUN and AKT.

## 1 Introduction

Polycystic ovary syndrome (PCOS) is among the most common gynecological endocrine diseases affecting the health of women of childbearing age. Globally, the prevalence of PCOS ranges from 4% to 21% (Bozdag et al., 2016). Clinical symptoms of PCOS include menstrual irregularities, infertility, hirsutism, acne, obesity, acanthosis nigricans, and mood disorders (Legro et al., 2013). Clinically, combined oral contraceptives are the first-line treatment for PCOS menstrual abnormalities (Mohamed et al., 2018), hirsutism, and acne (Frey and Aakvaag, 1981). Clomiphene is used as a first-line drug for the treatment of PCOS infertility (Misso et al., 2012). However, its efficacy is often limited and associated with many side effects (Hughes et al., 2010). As PCOS is a heterogeneous disease with different complexities and individual patient manifestations, the clinical treatment and comprehensive management of PCOS are extremely challenging (Christ and Cedars, 2023).

Moreover, most patients with PCOS seek medical treatment in adolescence or early childbearing age for irregular menstruation, acne, and infertility (Witchel et al., 2020). Most treatments focus on treating current clinical symptoms while ignoring the long-term health risks associated with PCOS. Therefore, developing a more complete treatment plan for PCOS is crucial. Recently, several studies have exhibited that traditional Chinese medicine can improve PCOS with fewer adverse reactions (Moini Jazani et al., 2019).

Single-cell RNA sequencing (scRNA-seq) is used to directly analyze gene expression, intracellular population heterogeneity, and cell status at the single-cell level. This can help us better understand transcriptional dynamics and gene regulatory relationships (Azizi et al., 2018). The Cangfu Daotan Decoction (CFDT) is an herbal formula consisting of ten medicinal herbs (the main components of CFDT are displayed in Table 1). It exhibits a significant effect in treating obesity infertility; however, its potential targets and mechanisms of action remain unclear. Network pharmacology is an emerging discipline that comprehensively uses multidisciplinary knowledge (Alam et al., 2022), such as systems biology, multi-omics, pharmacology, and computer network analysis, to study the global, systematic, and network nature of drug effects (Zhao et al., 2023). Recently, network pharmacology has provided new research methods to reveal the complex mechanisms of traditional Chinese medicine. Our study used scRNA-seq technology to identify differentially expressed genes in immune cells in the peripheral blood of patients with PCOS, we systematically analyzed the characteristics of the immune microenvironment. We used network pharmacology and molecular docking technology to systematically analyze the possible active ingredients and potential molecular interaction mechanisms of CFDT in treating PCOS to provide a scientific basis for the pharmacological research and clinical treatment of CFDT in PCOS treatment. Furthermore, we explored the clinical efficacy of CFDT in treating PCOS, particularly in patients with obesity.

**TABLE 1:** Composition and action of CFDT.

Pinyin name	Full species name	Pharmacological effects	Toxicity	Doses (g)
Cangzhu	*Atractylodes lancea (Thunb.) DC.* [*Asteraceae; Atractylodis Rhizoma*]	Cangzhu has anti-inflammatory, stimulates intestinal peristalsis, regulates gut microbiota, and improves cholesterol metabolism (Koonrungsesomboon et al., 2014; Qu et al., 2022).	NA (Liu et al., 2008)	15
Xiangfu	*Cyperus rotundus L.* [*Cyperaceae; Cyperi Rhizoma*]	Xiangfu has anti-androgenic, anti-diabetic, anti-inflammatory, lipid-lowering, and anti-obesity effects, and it improves lipid and glucose metabolism (Kanagali et al., 2022; Pirzada et al., 2015; Xue et al., 2023).	NA (Abo-El-Yazid et al., 2022)	10
Banxia	*Pinellia ternata (Thunb.) Breit.* [*Araceae; Pinelliae Rhizoma*]	Banxia has anti-inflammatory, anti-tumor, antitussive, and antiasthmatic properties (Tao et al., 2023).	Irritant toxicity, cardiotoxicity, hepatotoxicity and embryotoxicity, but the toxicity is weakened after processing (Zou et al., 2023)	9
Chenpi	*Citrus reticulata Blanco* [*Rutaceae; Citri Reticulatae Pericarpium*]	Chenpi can improve hepatic steatosis, oxidative stress, and inflammation (Ke et al., 2020).	NA (Nakajima et al., 2020)	10
Fuling	*Wolfiporia cocos (Schwein.) Ryvarden and Gilb.* [*Polyporaceae; Poria*]	Fuling has antibacterial, antioxidant, anti-inflammatory, immunomodulatory, and hepatoprotective and renoprotective effects (Nie et al., 2020).	NA (Li et al., 2022)	15
Zhiqiao	*Citrus aurantium L.* [*Rutaceae; Aurantii Fructus Immaturus*]	Zhiqiao has anxiolytic, anti-obesity, antibacterial, antioxidant, insecticidal, and anti-diabetic activities (Suntar et al., 2018).	NA (Lamichhane et al., 2022)	12
Chuanxiong	*Ligusticum chuanxiong Hort.* [*Apiaceae; Chuanxiong Rhizoma*]	Chuanxiong has anti-inflammatory, antioxidant, neuroprotective, and antifibrotic effects (Ran et al., 2011).	NA (Long et al., 2023)	6
Dannanxing	*Arisaema erubescens (Wall.) Schott* [*Araceae; Arisaematis Rhizoma Praeparatum*]	Dannanxing can dry dampness, resolve phlegm, dispel wind, relieve pain, and reduce swelling (Wang et al., 2023b).	Nephrotoxicity, toxicity is reduced after processing (Wang et al., 2023b)	6
Huashi	*Talc*	Huashi has antibacterial and adsorptive effects (Addala et al., 2022).	Inhalation toxicity (Johnson, 2021)	6
Shenqu	*Massa Medicata Fermentata*	Shenqu can improve intestinal flora and fight inflammation (Zhang et al., 2020).	NA (Liu et al., 2022)	8

## 2 Materials and methods

**FIGURE 1:**
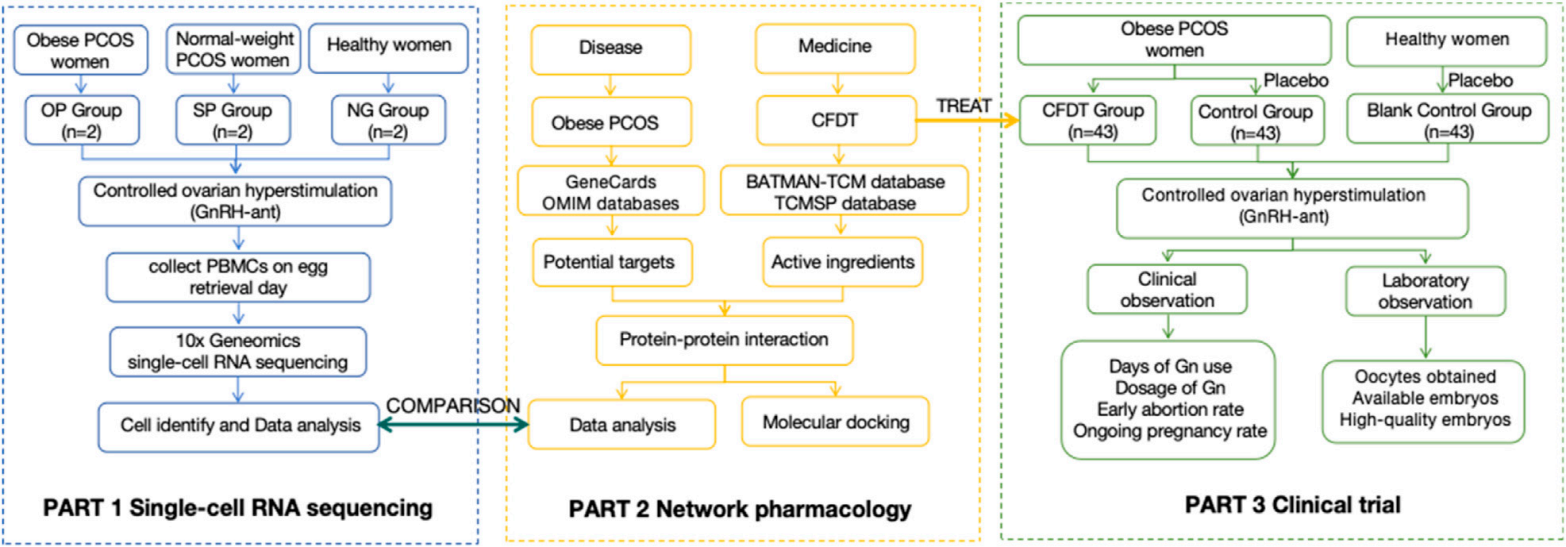
Flow chart of the study.

This study was divided into three parts. In part 1, we used scRNA-seq to analyze the immune microenvironment status of patients with PCOS. In part 2, we used network pharmacology to analyze the targets of CFDT. In part 3, we explored the clinical effect of CFDT based on a double-blind clinical trial. This process is illustrated in Figure 1.

### 2.1 Diagnostic criteria

The diagnostic criteria for PCOS were formulated following Rotterdam criteria in 2003 (2 out of 3) (Rotterdam, 2004):

1) Oligo- or anovulation,
2) Clinical and biochemical signs of hyperandrogenism,
3) Polycystic ovaries.

### 2.2 Inclusion criteria

Patients with PCOS met the diagnostic criteria for PCOS and the following conditions:

1) Age: 22–40 years,
2) Cohabitation for more than 1 year after marriage, normal sexual life, no contraception, and no pregnancy,
3) The husbands’ semen analysis results were generally normal.

Obese patients with PCOS have a body mass index (BMI) > 30 kg/m^2^; normal-weight patients with PCOS have a BMI <23 kg/m^2^ (Caballero, 2019).

The following conditions were met for healthy women:

1) Age: 22–40 years old,
2) Cohabitation for more than 1 year after marriage, normal sexual life, no contraception, and no pregnancy.

### 2.3 Exclusion criteria

1) Those who could not get pregnant due to reproductive system malformation or severe systemic diseases,
2) Those with pelvic inflammation, tubal adhesion, obstruction, and adrenal cortical insufficiency,
3) Those with autoimmune diseases such as systemic lupus erythematosus, rheumatoid arthritis, and inflammatory bowel disease,
4) Those with infertility caused by other diseases, such as endometriosis and hyperprolactinemia,
5) Those who did not meet the inclusion criteria did not take medication as prescribed or had poor compliance.

### 2.4 scRNA-seq

In part 1, obese patients with PCOS (OP group), non-obese patients with PCOS (SP group), and women with simple male factor infertility (NG group) who underwent *in vitro* fertilization-embryo transfer (IVF-ET) at the Reproductive and Genetic Center of our hospital and met the inclusion criteria were selected. Peripheral blood was collected from each subject on the day of egg retrieval. All patients were subjected to controlled ovarian hyperstimulation with antagonist protocol. When there were more than three follicles with a diameter ≥18 mm, 8,000–10000 IU of human chorionic gonadotropin (hCG) was injected intramuscularly to induce follicle maturation, and transvaginal oocyte retrieval was performed 36 h later (Reichman et al., 2011). Peripheral blood mononuclear cells (PBMCs) were separated by density gradient centrifugation (Golke et al., 2022). High-throughput sequencing was performed using the 10x Geneomics sequencing platform to obtain sequencing data for subsequent data analysis. CellRanger software was used to control the data quality. Barcode sequence markers in the sequence and UMI markers of different mRNA molecules in each cell were identified for quantification to obtain quality control statistical information, such as the number of high-quality cells and gene median. Principal component analysis was performed using gene expression, and uniform manifold approximation and projection (UMAP) dimensionality reduction technology was used for visual clustering in two-dimensional images (Yang et al., 2021).

### 2.5 Cell definition and differential gene analysis

**FIGURE 2:**
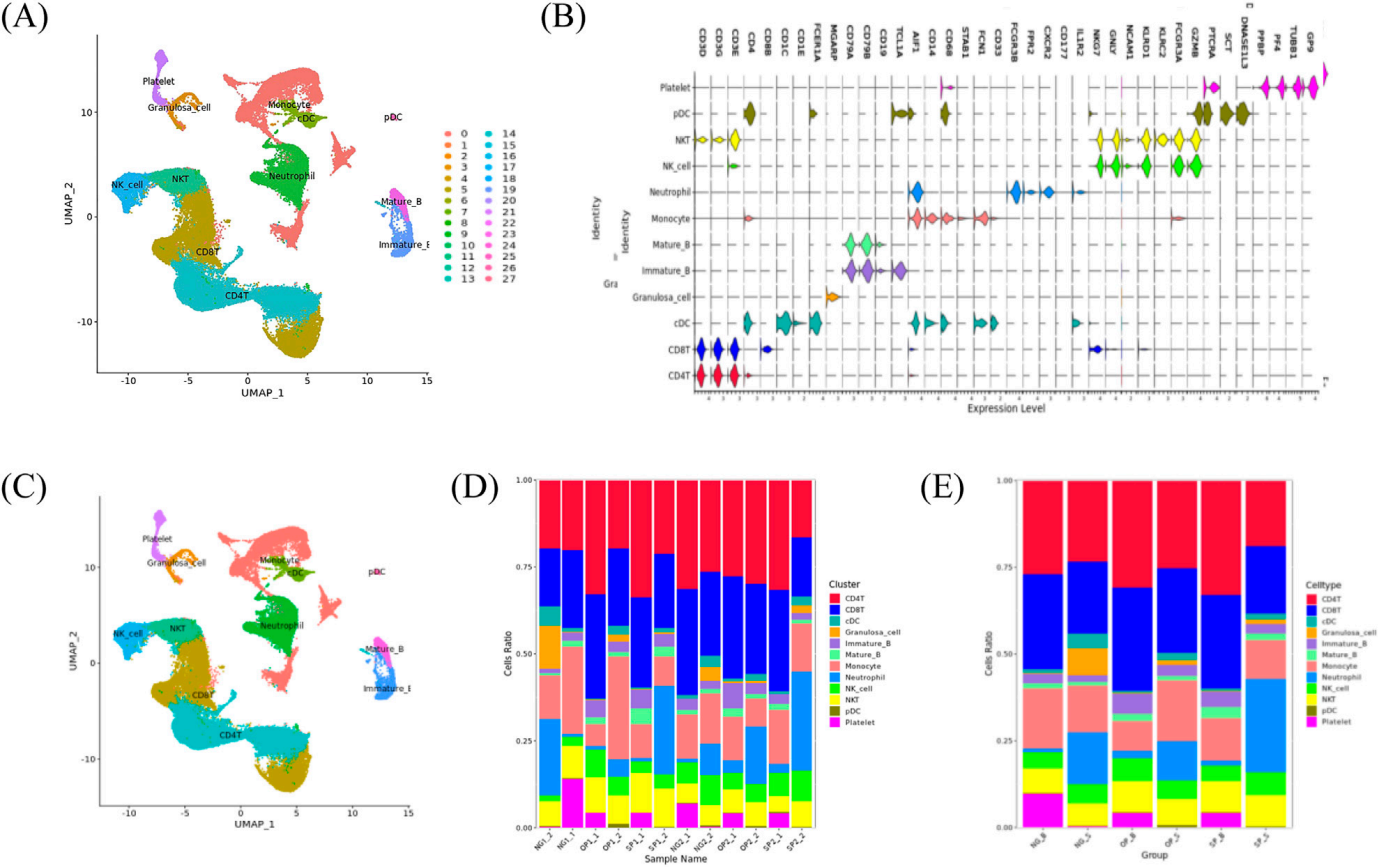
ScRNA-seq cell definition results. **(A)** Cell clustering UMAP visualization clustering results. **(B)** Violin plot of marker gene expression in each cluster. **(C)** UMAP plot of the cell subpopulation distribution. **(D)** Histogram of the cell proportion in each sample. **(E)** Histogram of the cell proportion in each sample.

The SingleR package was used to calculate the Spearman’s correlation between the cell expression profile to be identified, and the reference dataset based on the single-cell reference expression quantification public dataset, and the cell type that had the greatest correlation with the sample cells was selected as the final cell type to be identified (Figure 2). According to *P* < 0.05 and the difference fold |log _2_ FC|>0.25, the significantly different genes in obese patients with PCOS were screened out, and the gene ontology (GO) analysis of the differential genes and the Kyoto encyclopedia of genes and genomes (KEGG) were performed through the hypergeometric distribution test. KEGG and Reactome analyses were used to identify GO functional items with significantly enriched differential genes and the main metabolic and signal transduction pathways involved.

### 2.6 Collection of chemical components and target prediction of CFDT

In part 2, the traditional Chinese medicine database and analysis platform (TCMSP, https://tcmsp-e.com/) was used to retrieve the classic prescriptions used in this study and the constituent Chinese medicines of CFDT. The BATMAN-TCM database (http://bionet.ncpsb.org.cn/batman-tcm/) was used for Chinese medicines that could not be retrieved by the TCMSP database. The TCMSP database was used to retrieve the active ingredients of *Atractylodes lancea*, *Cyperus rotundus*, *Pinellia ternata (Thunb.) Breit.*, *Citrus reticulata Blanco*, *Poria*, *Citrus aurantium*, and *Ligusticum chuanxiong*. Oral bioavailability ≥30% and drug-likeness (DL) ≥ 0.18 were used as screening conditions to obtain the possible active ingredients of the compound. By logging into the BATMAN-TCM database (http://bionet.ncpsb.org.cn/batman-tcm/), the effective ingredients of *Arisaema consanguineum*, *Talc*, and *Massa Medicata Fermentata* and proteins with high credibility were obtained as candidate genes. The targets obtained were calibrated using data from the UniProt database (https://www.uniprot.org/), non-human genes were eliminated, invalid duplicate targets were deleted, and standardized gene names were obtained. Using “obese polycystic ovary syndrome” as the keyword, GeneCards (https://www.genecards.org/) and OMIM (https://www.omim.org/) databases were used to search to obtain possible targets related to the disease. All targets in the database were integrated into a table, duplicate genes were eliminated, and the UniProt database was used for correction to obtain disease-target gene information. To further study the protein-protein interaction of CFDT in the treatment of obese PCOS, the drug-intersection genes were uploaded to the interaction database String (https://string-db.org/) was used to construct the protein-protein interaction network (PPI), and the core targets were selected to make the protein interaction network diagram. Cytoscape is an information visualization software primarily used to visually analyze biological information and social networks. Cytoscape (version 3.7.2) was used to draw the CFDT network diagrams and a common target PPI network diagram. The network was topologically analyzed, with the node size and color reflecting the degree value. The larger the node, the greater the degree value. The thickness of the edge was used to reflect the size of the combined score. The thicker the edge, the greater the combined score. The higher the degree value of a node, the higher its degree of centrality, which means that the node is more important in the network (Udrescu et al., 2020). The clusterProfiler package of Rstudio was used to perform GO enrichment and KEGG pathway analyses on the target genes (Chen et al., 2022). The structure files of the key active ingredients of CFDT were retrieved through the PubChem database (https://pubchem.ncbi.nlm.nih.gov/). The receptor proteins were retrieved from the Protein Data Bank (http://www.rcsb.org/pdb) database. The receptor proteins were molecularly docked with ligand small molecules using AutoDock Vina software (version 1.1.2). The affinity was evaluated and visualized according to the spatial effect, repulsion, and hydrogen bond of the receptor-ligand complex.

### 2.7 Trial drugs and schemes

The cases included in part 3 were from the Integrative Medicine Research Center of Reproduction and Heredity in Affiliated Hospital of Shandong University of Traditional Chinese Medicine (from June 2021 to June 2023). In part 3, all obese patients with PCOS who underwent IVF treatment were selected and divided into the CFDT and control groups using the random number table method (43 cases in each group). Forty-three normal women whose husbands were infertile were selected as the blank control group. Serum luteinizing hormone, follicle-stimulating hormone, and estradiol levels were measured on the third day of the menstrual cycle before entering the cycle, and antral follicle count was measured by transvaginal ultrasound. The subjects were administered CFDT or placebo (both provided by the Preparation Room of the Affiliated Hospital of Shandong University of Traditional Chinese Medicine, mainly composed of dextrin, 3 g/packet) for intervention according to the groups, three packets at a time, twice a day, starting from the sixth day of menstruation (two cycles before IVF cycles), stopping during menstruation until the hCG administration day of the IVF cycle. Peripheral blood serum was collected on the day of egg retrieval.

The decoction was prepared as follows: 1) Talcum (8 g) and Massa Medicata Fermentata (15 g) were wrapped separately in gauze and steeped together with the other eight herbs in cold water for 30 min 2) The mixture was boiled and simmered for 30 min before pouring the liquid. 3) Cold water was added again, and the mixture was boiled for another 20 min, after which the liquid was poured out. 4) The decoctions from both the boiling sessions were combined. Strain out the residue. The resulting decoction was divided into two doses to be administered daily, with each dose of 150 mL.

### 2.8 Statistical analysis

The original database was established using Excel, and the data were statistically analyzed using the Statistical Package for the Social Sciences software (version 23.0). If the data met normality, variance analysis was used to compare the three groups, and *P* < 0.05 indicated that the difference was statistically significant. The measurement data are expressed as the mean ± standard deviation (¯x ± s). The comparison of the rates between the two groups was performed using the Fisher exact probability method of the four-cell table data using the χ^2^ test, and *P* < 0.05 indicated that the difference was statistically significant.

## 3 Results

### 3.1 Identification of cell clusters and dimension reduction analysis

The scRNA-seq analysis identified 27 cell clusters and seven cell types. Marker gene expression in the eight-cell types differed between cell types. The proportions of T cells, B cells, and natural killer (NK) cells in patients with PCOS were higher than those in normal women, with even higher proportions observed in obese patients with PCOS. The proportion of DC and monocytes in patients with PCOS was lower than in normal women. There was a non-significant difference in the proportion of DC cells between OP and SP groups. Compared with the SP group, the OP group had a more significant decrease in monocytes.

Pairwise comparisons were made between NG and OP, NG and SP, and SP and OP groups, and the differences related to obesity and PCOS were screened (*P* < 0.05, |log_2_ FC|>0.25). The clusterProfiler package was used to perform GO and KEGG enrichment analyses on the differentially expressed genes of each cell group in NG_B and OP_B groups. The B cell KEGG enrichment results were non-significant; therefore, we used reactome enrichment analysis instead.

### 3.2 CD4^+^ T cell activation in obese PCOS

**FIGURE 3:**
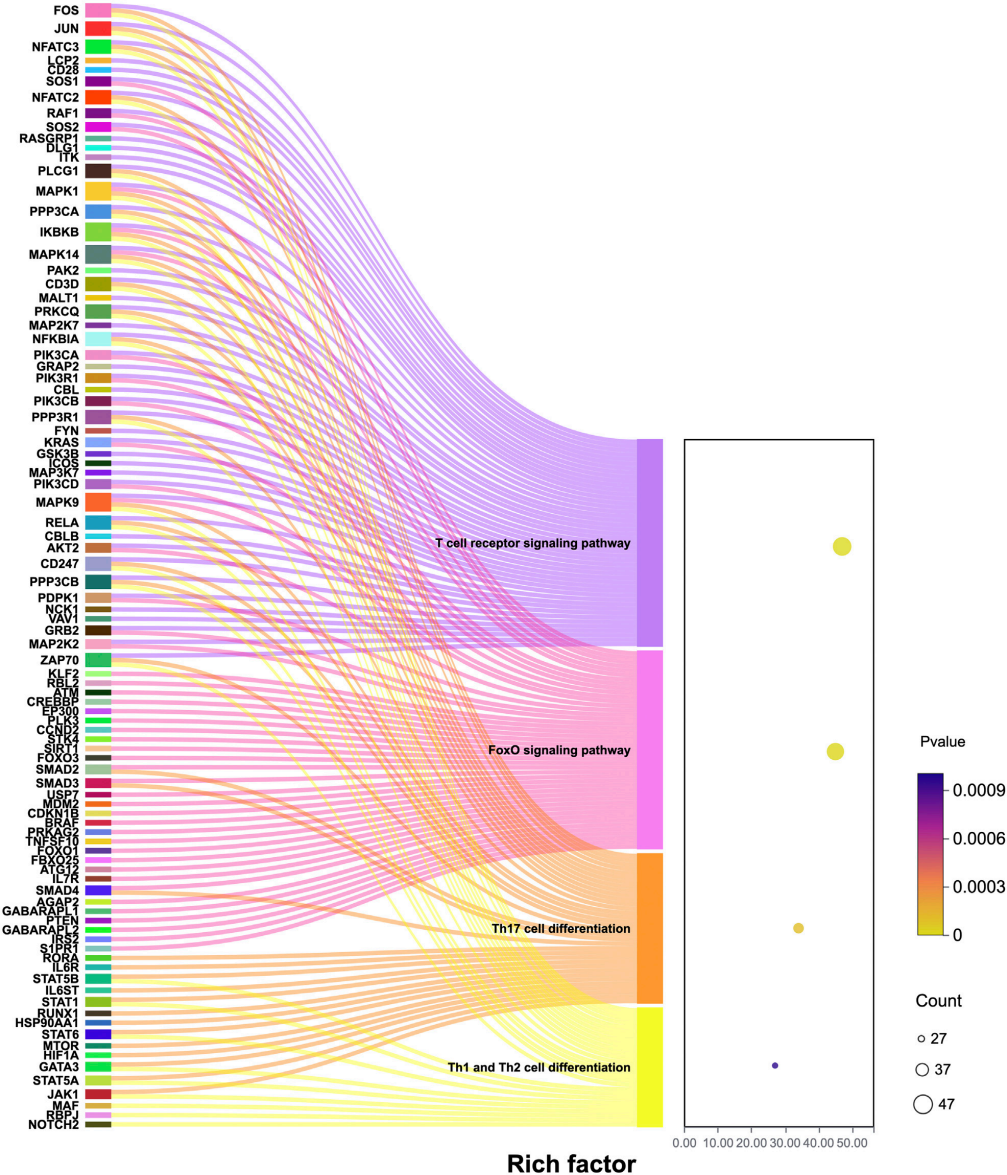
Differential gene enrichment mulberry bubble chart.

Compared with normal women, the CD4^+^ T cell activation-related genes JUN, NFATC2, SOS1, AKT, and MAPK9 in patients with PCOS and obesity were upregulated and enriched in the T cell receptor (TCR) signaling pathway, FoxO signaling pathway, Th17 cell differentiation signaling pathway, and Th1 and Th2 cell differentiation pathways, as depicted in Figure 3. The TCR signaling pathway is a critical part of the immune system (Shah et al., 2021), is mainly responsible for the activation and functional regulation of T cells, and is essential for T cells to recognize antigens, produce immune responses, and maintain the balance of the immune system (Rellahan et al., 1997). JUN is at the “crossroads” of the TCR signaling pathway and is a key gene in the TCR signaling pathway (Ono, 2020).

**FIGURE 4:**
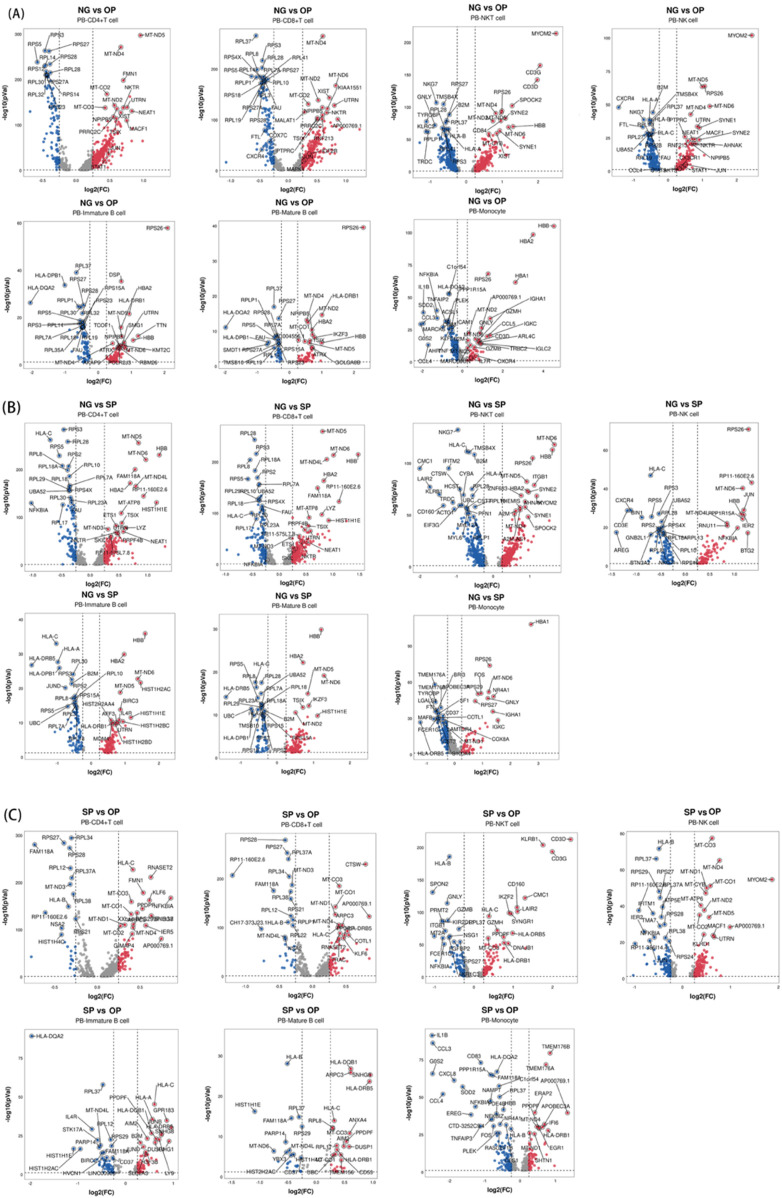
Volcano plot of differentially expressed genes in PBMCs between each group. **(A)** Differential genes between NG and OP. **(B)** Differential genes between NG and SP. **(C)** Differential genes between SP and OP.

In this study, we analyzed the differentially expressed genes between NG and OP and NG and SP groups (*P* > 0.05, |log_2_ FC| ≥ 0.25) (Figure 4). A total of 19 genes were screened, including CYLD, ARPC3, CXCR4, RORA, JUN, FGL2, ZEB2, GNLY, FTL, SMAD3, interleukin (IL)7R, KIR2DL1, CTSD, BTG2, CCL5, HLA, RETN, CTSZ, and NCF2. These potential target genes are related to Th1, Th2, Th17 differentiation, FγR-mediated phagocytosis pathway, NOD-like receptor signaling pathway, NF-κB signaling pathway, FoxO signaling pathway, and tumor necrosis factor (TNF) signaling pathways, MHC protein complex binding, antigen presentation function, ferroptosis, and oxidative phosphorylation.

### 3.3 Prediction of candidate bioactive ingredients for CFDT treatment of obese PCOS

**FIGURE 5:**
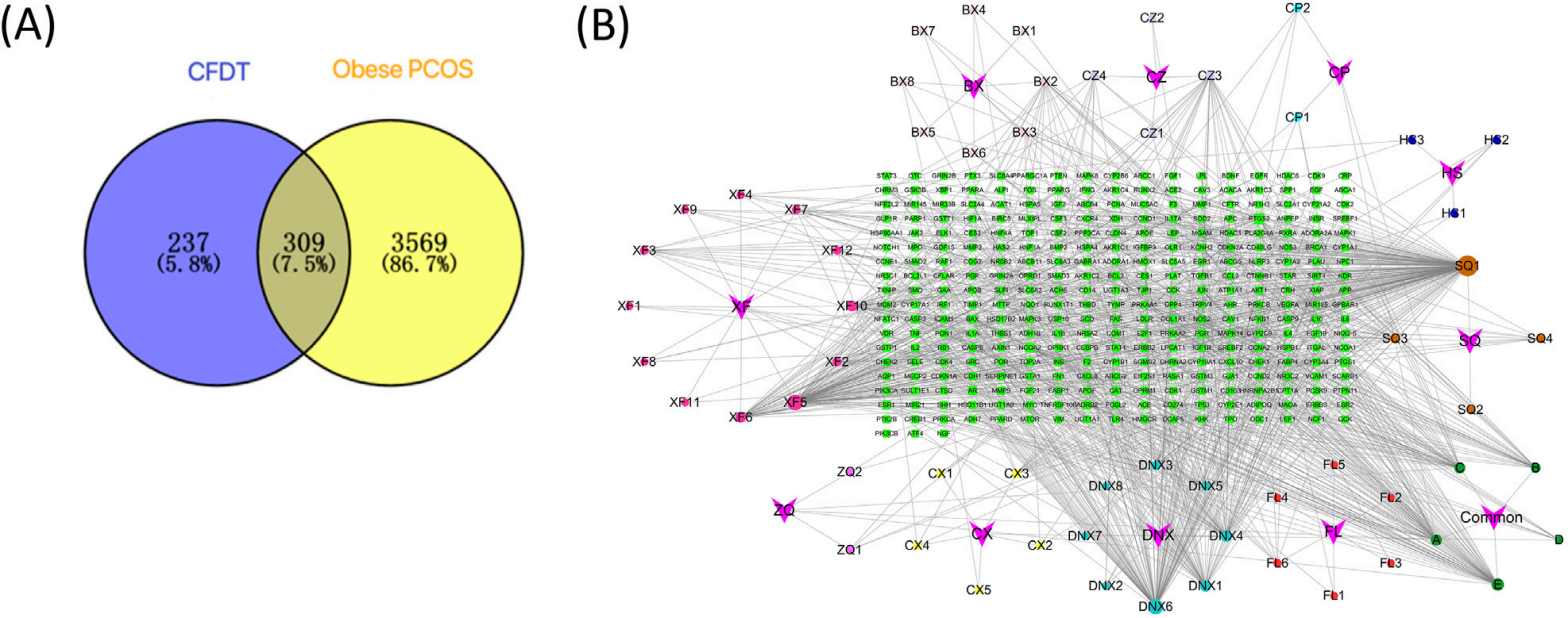
**(A)** Venn diagram of CFDT ingredients and potential targets of obese PCOS. **(B)** CFDT active ingredient-target network.

According to the TCMSP and BATMAN-TCM databases, screening by OB ≥ 30% and DL ≥ 0.18, 56 candidate bioactive ingredients were identified (Table 1). Furthermore, using the GeneCards and OMIM databases, 3,308 and 646 small molecules were obtained, respectively. After deduplication, 3,878 targets related to obese PCOS were identified and the obtained genes were corrected using the UniProt database. The intersection of active ingredients and disease targets is depicted in a Venn diagram (Figure 5A). Finally, the direct active ingredient-target interaction network of CFDT was constructed using Cytoscape (Figure 5B). These genes may act as interactive target genes for treating obese PCOS. The top five candidate bioactive ingredients (with high degree value) are quercetin, apigenin, β-sitosterol, cholesterol, and nobiletin.

**FIGURE 6:**
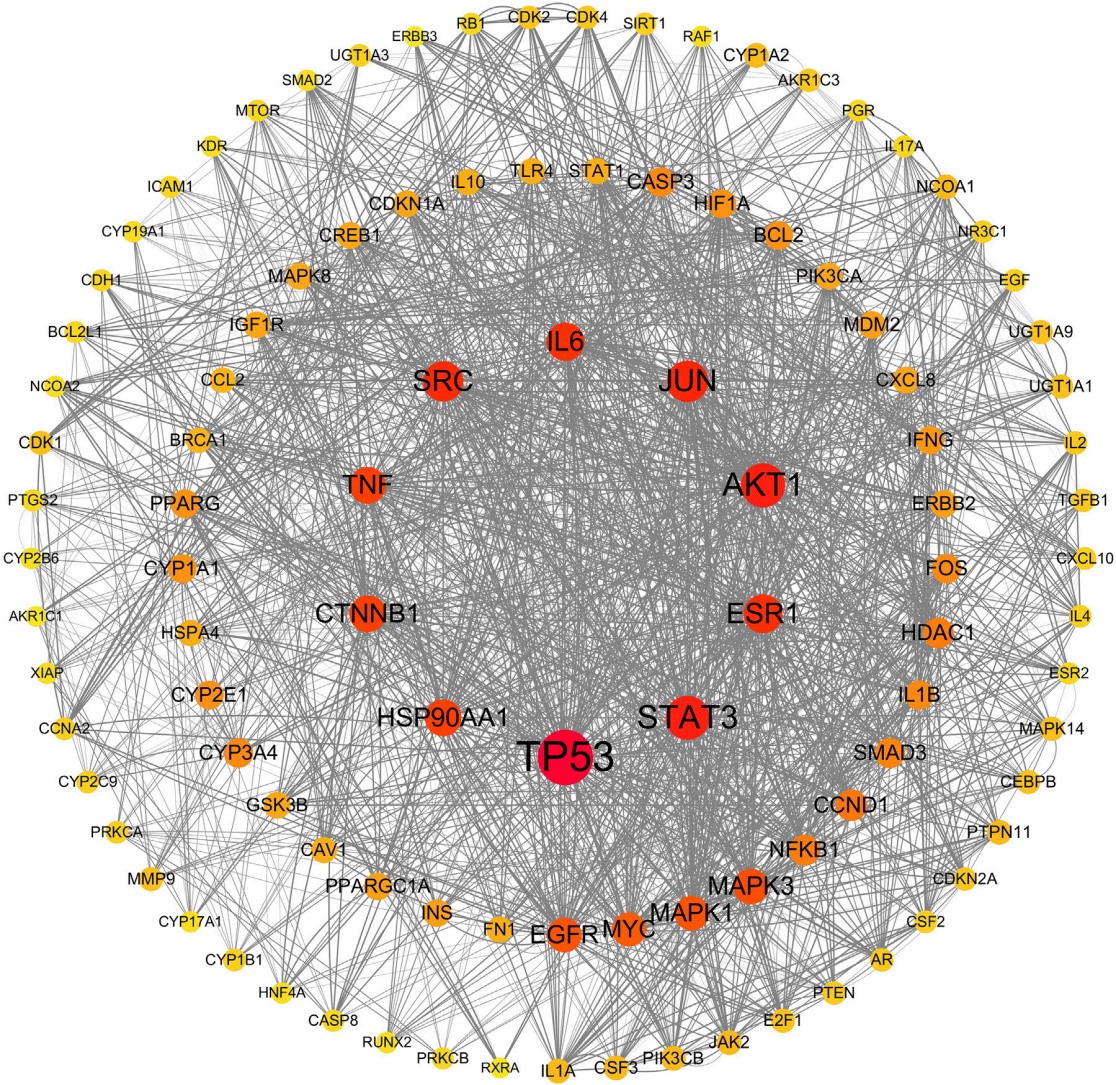
PPI network of genes for CFDT treatment of obese PCOS.

All 309 genes in the gene set for CFDT treatment of obese PCOS were imported into STRING, and the PPI network of genes for CFDT treatment of obese PCOS was constructed using Cytoscape (version 3.7.2), as revealed in Figure 6. The key targets in PPI network were tumor protein 53 (TP53), the serine/threonine kinase AKT1 (AKT1), signal transducer and activator of transcription 3 (STAT3), JUN (Jun proto-oncogene, Jun), sarcoma gene (SRC), estrogen receptor 1, interleukin 6, heat shock protein 90 alpha family class a member 1, TNF, and catenin beta 1.

**FIGURE 7:**
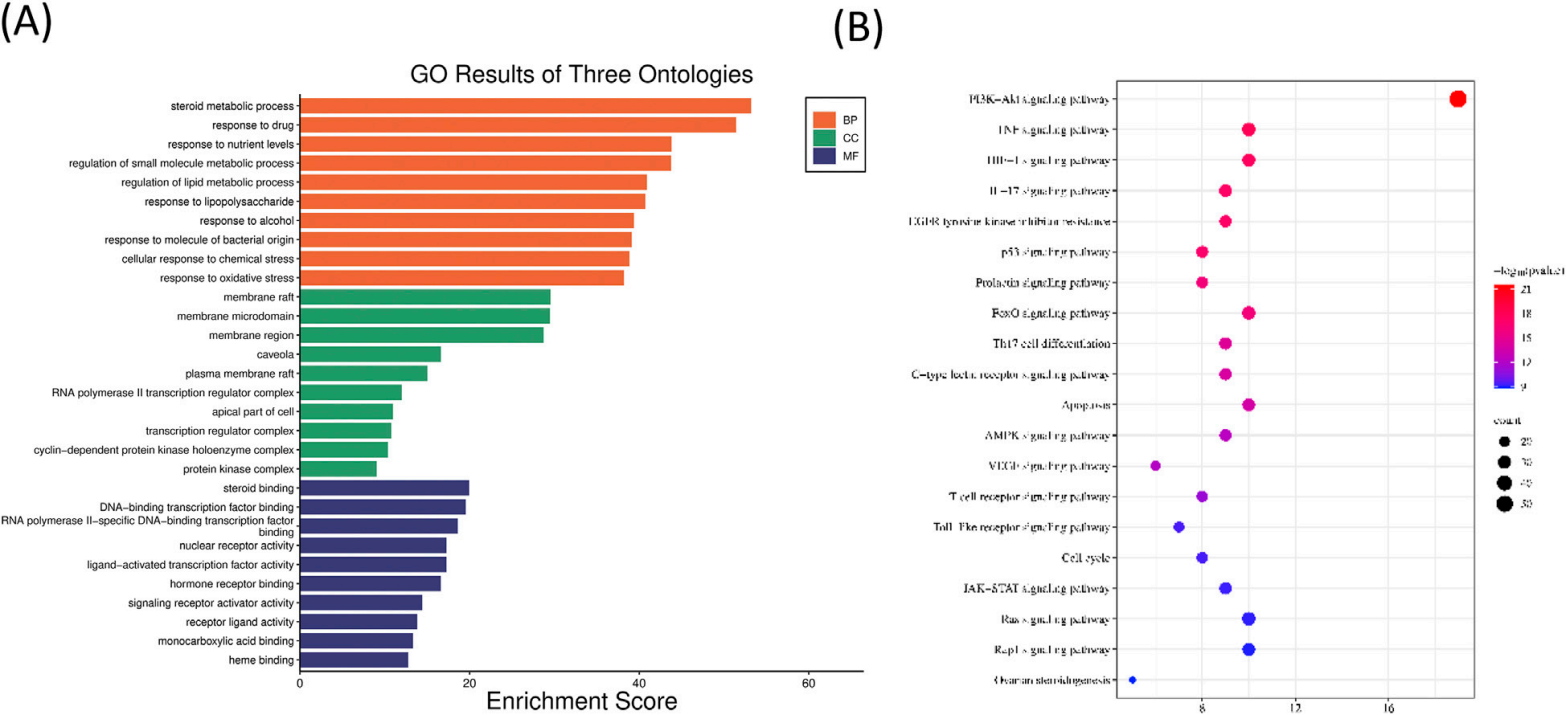
**(A)** GO analysis of the CFDT treatment of obese PCOS. **(B)** KEGG analysis for CFDT treatment of obese PCOS.

According to the GO analysis with the lowest *P* value, the top five biological processes are steroid metabolic process, response to drug, response to nutrient levels, regulation of small molecule metabolic process, and regulation of lipid metabolic process. The top five cellular components are membrane raft, membrane microdomain, membrane region, caveola, and plasma membrane raft. The top five molecular functions are steroid binding, DNA-binding transcription factor binding, RNA polymerase II-specific DNA-binding transcription factor binding, nuclear receptor activity, and ligand-activated transcription factor activity (the bar graph of the top 10 is displayed in Figure 7A). The results of KEGG functional enrichment analysis revealed that the active components of CFDT affected obese PCOS mainly through the PI3K-AKT, TNF, HIF-1, and IL-17 signaling pathways, and EGFR tyrosine kinase inhibitor resistance (the bubble chart of the top 20 is exhibited in Figure 7B).

### 3.4 Molecular docking of the active components of CFDT with core targets

**FIGURE 8:**
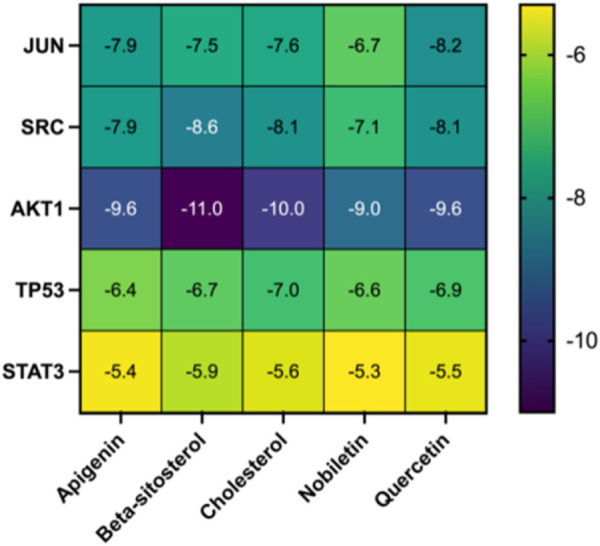
Heatmap of the molecular docking score. Binding energy (kcal/mol) of active compounds of herbs and key target molecules.

Molecular docking was used to assess the efficacy of the screened active drugs and targets to validate the outcomes of network pharmacology. The five compounds with the highest degree values in the herb-compound-target network analysis and the key targets (TP53, AKT1, STAT3, JUN, and SRC) based on the degrees of common targets in the PPI network were selected for molecular docking by PPI network screening using AutoDock Vina (version 1.1.2). The results depicted that all five critical active ingredients exhibited good binding activity with all five core targets (Figure 8). JUN and AKT1 have relatively lower binding energies with the five vital active ingredients; therefore, the effect is the best, as depicted in Figure 8 and Table 2.

**TABLE 2:** Docking patterns of active compounds and key targets.

	AKT1	JUN
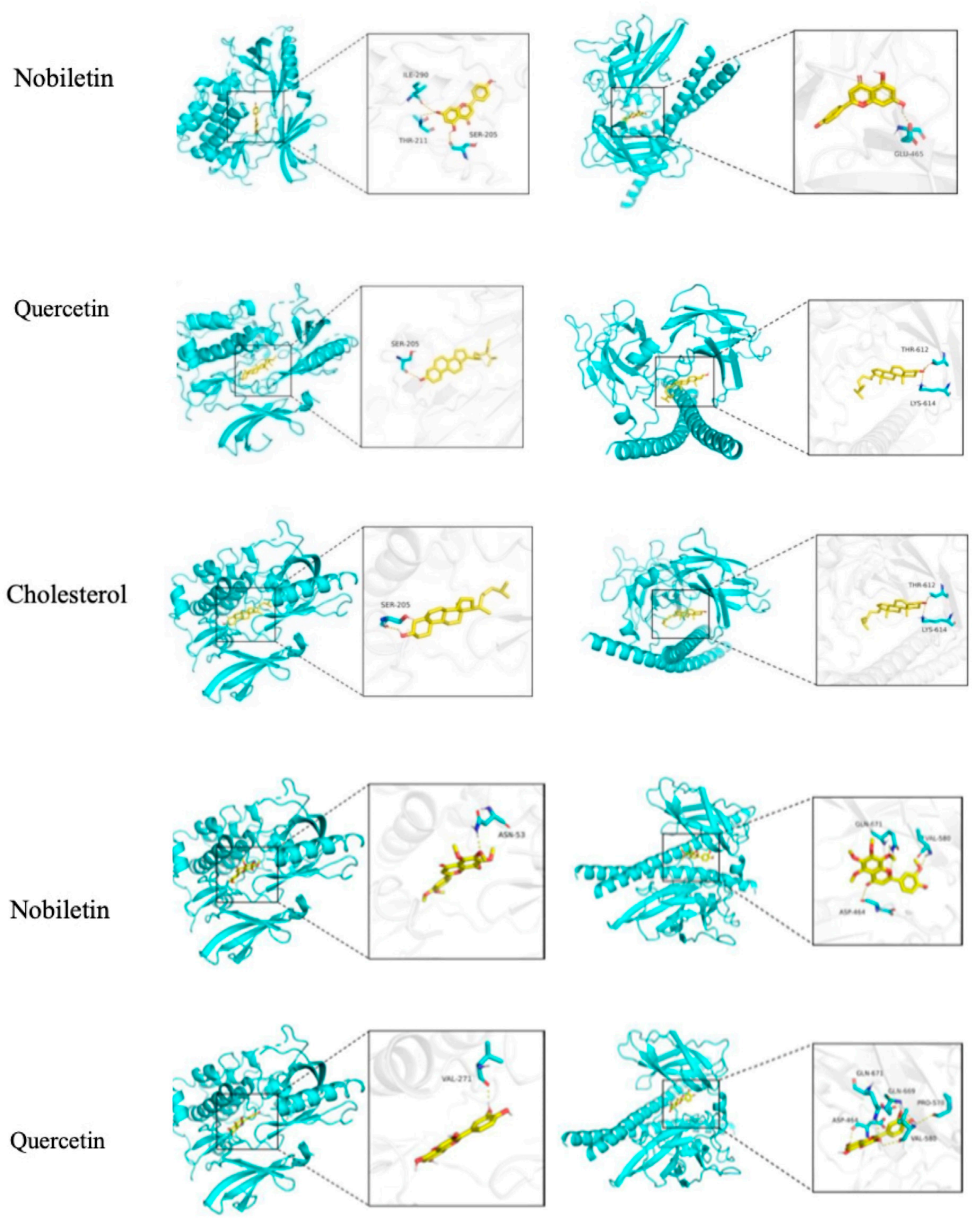		

### 3.5 Comparison of clinical manifestations and laboratory outcomes

In part 3, the gonadotropin (Gn) dosage, Gn medication days, early abortion rate, ongoing pregnancy rate (Table 3), number of oocytes obtained, number of available embryos, and number of high-quality embryos were compared among the three groups of patients (Table 4).

**TABLE 3:** Comparison of clinical manifestations among three groups of patients.

Group	Gn medication days	Gn dosage (IU)	Early abortion rate	Ongoing pregnancy rate
blank control group (n = 41)	8.98 ± 2.21	1786.89 ± 406.28	2/41 (9.76%)	19/41 (46.3%)
CFDT group (n = 40)	8.75 ± 1.43	1896.56 ± 336.90^#^	6/40 (15%)	16/40 (40.0%)^#^
control group (n = 38)	9.82 ± 1.67	2260.53 ± 586.67*	5/38 (13.2%)	6/38 (15.8%)*

*P ˂ 0.05 compared with blank control group.

^#^ *P* ˂ 0.05 compared with control group.

**TABLE 4:** Comparison of laboratory outcomes among three groups of patients.

Group	Oocytes obtained	Available embryos	High-quality embryos
blank control group (n = 41)	9.27 ± 4.25^#^	8.39 ± 4.19^#^	3.71 ± 1.47
CFDT group (n = 40)	21.03 ± 6.61*	17.33 ± 2.61*^#^	3.83 ± 1.38^#^
control group (n = 38)	23.79 ± 5.82*	20.00 ± 4.56*	2.76 ± 1.30*

*P ˂ 0.05 compared with blank control group.

^#^ *P* ˂ 0.05 compared with control group.

## 4 Discussion

The symptoms of PCOS usually start with puberty and are characterized by excessive androgen, irregular ovulation, and polycystic ovarian changes. It usually coexists with insulin resistance, dyslipidemia, and obesity (Luan et al., 2022). It also has a significant risk of cardiovascular and metabolic sequelae (including diabetes and metabolic syndrome). It is among the most common gynecological endocrine diseases that affect the health of women of childbearing age (Meier, 2018; Sadeghi et al., 2022). It is now generally believed that PCOS is closely linked to immune dysregulation and chronic low-level inflammation (Wang J. et al., 2023). Therefore, treatment from an immune perspective may become a new approach to treat PCOS. Traditional Chinese medicine has exhibited certain advantages in clinical practice, but there is a lack of high-quality research evidence. Consequently, we focused on an immunological perspective, using scRNA-seq and network pharmacology technology to explore the possible mechanism of traditional Chinese medicine in treating PCOS, specifically obese PCOS (Moini Jazani et al., 2019).

Changes in the proportion of immune cells are a key sign indicating a change in the immune microenvironment. In our study, the T cell proportion in peripheral blood samples of obese patients with PCOS increased. From the perspective of cell cluster gene set expression, the T cell function of obese patients with PCOS was in a relatively stimulated state compared with that of the NG group. The differential gene enrichment results of T cells in obese patients with PCOS were mainly concentrated in biological processes, such as protein localization, immune defense, viral infection, cell adhesion, and immune regulation, which are closely associated with the immune response. KEGG enrichment analysis mainly focused on the TCR signaling pathway, antigen processing and presentation, Th-17 signaling pathway, NF-κB signaling pathway, FoxO signaling pathway, B-cell receptor signaling pathway, Th17 cell differentiation pathway, and Th1 and Th2 cell differentiation pathways. These signaling pathways are closely correlated with T-cell activation, differentiation, and cytokine release (Shah et al., 2021). The TCR signaling pathway is crucial for T cell activation and is an important signaling pathway in the immune response (Alcover et al., 2018). It is also involved in antigen recognition, cytokine production, immune responses, and other processes. Furthermore, the potential target genes CYLD (Reiley et al., 2007) and CXCR4 in the T cells of obese patients with PCOS are crucial for maintaining T cell homeostasis.

In this study, we used network pharmacology to construct the intersection target network diagram of CFDT and GO and KEGG enrichment analysis to reveal the core targets and signaling pathways of CFDT in treating obese PCOS. Fifty-six effective active ingredients of CFDT were obtained, 3,878 genes related to obese PCOS were obtained, and 309 potential drug targets were obtained, of which TP53, AKT1, STAT3, JUN, and SRC were the top five targets. These potential target genes are vital for immune and inflammatory processes. They participate in regulating immune and inflammatory responses through different mechanisms and pathways.

TP53 is a tumor suppressor gene that encodes the p53 protein, which plays a key role in cell cycle regulation, DNA repair, cell apoptosis, and tumor inhibition. p53-mediated GHRH antagonists have a positive effect on inflammation (Barabutis et al., 2018). AKT1 is a member of the AKT family. The AKT signaling pathway regulates the survival, differentiation, and activation of immune cells while affecting the inflammatory response. AKT activation can promote the survival and function of T and B cells and produce inflammatory factors (Santinon et al., 2022; Szydlowski et al., 2014; Zhao et al., 2019). STAT3 is a key factor in the JAK-STAT signaling pathway and is involved in cell growth, differentiation, survival, and immune regulation (Hillmer et al., 2016). Studies have found that STAT3 can inhibit autocrine IFN signaling in mouse DC and regulate Th17/Treg cell homeostasis (Zhao et al., 2021). The gene JUN which overlaps with the results of Part 1 is a major component of the AP-1 transcription factor complex and can directly participate in inflammatory responses and immune regulation by regulating the expression of inflammatory factors and cytokines (Hata et al., 2023). When T cells recognize and bind to antigenic peptides of the major histocompatibility complex through their T cell receptors, the TCR signaling pathway is activated. In the TCR signaling pathway, AP-1 is regulated by multiple upstream signals, including a series of phosphorylation events triggered by Src family kinases, Syk family kinases ZAP-70, and intrachain kinases such as Lck and Fyn. These events promote signal transduction and activation pathways, such as the Ras/MAPK pathway, leading to the activation of AP-1. AP-1 activation is essential for T cell function. It is directly involved in regulating the expression of multiple immune response-related genes, including cytokines, cell surface molecules, and factors that promote cell cycle progression. Thus, AP-1 plays a regulatory role in T cell proliferation, differentiation (such as differentiation of helper T cells Th1 and Th2), and cell death. SRC encodes a non-receptor tyrosine kinase. SRC kinases play a role in various cell types. SRC kinases regulate various key processes in the immune system, including cell activation, proliferation, differentiation, and cell-to-cell communication (Brian and Freedman, 2021). KEGG pathway enrichment analysis revealed that the potential targets of CFDT for obese PCOS were mainly immune regulation and inflammation-related signaling pathways, such as the IL-17 signaling pathway, FoxO signaling pathway, Th17 cell differentiation, TCR signaling pathway, PI3K-Akt signaling pathway, and Toll-like receptor signaling pathway. The immune system is a defense system composed of many biological structures that protect the host from diseases. If the human immune system is out of balance, it can lead to various diseases. Patients with PCOS were found to be in a chronic low-grade inflammatory state, including high white blood cell levels, endothelial dysfunction, and proinflammatory cytokine disorders (Gonzalez et al., 2014; Petrikova et al., 2010). It can be inferred that CFDT, a traditional Chinese decoction, may affect obese PCOS through mechanisms related to immune regulation and inflammatory responses.

In the clinical trial described in part 3, CFDT exhibited good clinical efficacy. Women of childbearing age usually release one egg during each menstrual cycle. To improve the success rate and reduce medical expenses, IVF patients will be treated using an ovarian stimulation superovulation protocol to obtain more eggs in a single cycle, thereby increasing the possibility of pregnancy. Studies have found that high doses of Gn during IVF-ET will affect the endocrine environment during egg growth and embryo transfer, which may affect embryo implantation and later development. Exogenous gonadotropins may also interfere with trophoblast growth and invasion, thereby affecting pregnancy outcomes (Liu et al., 2021). This study revealed that the doses of Gn used in the CFDT group (1896.56 ± 336.90 IU) were statistically lower than those in the control group (2260.53 ± 586.67 IU). The day of using Gn was also reduced. The dosage and duration of Gn use can reduce the economic and psychological burden on patients. The laboratory results indicated a statistically non-significant difference in the number of eggs retrieved between the CFDT (21.03 ± 6.61) and control groups (23.79 ± 5.82). However, the number of high-quality embryos in the CFDT group (3.83 ± 1.38) was statistically higher than in the control group (2.76 ± 1.30). This suggests that the CFDT group can obtain a more satisfactory number of eggs retrieved by reducing the dosage of Gn and improving D3 embryo quality. Clinical data revealed that the continuous pregnancy rate in the CFDT group (40%) was significantly higher than that in the control group (15.8%), indicating that CFDT can improve the treatment outcome of patients with obese PCOS.

Our study has some limitations. First, the sample size in part 1 was relatively small, which might have caused deviations in the results. In the future, more samples can be included in the scRNA-seq database for comprehensive analysis. Second, the study only focused on scRNA-seq, network pharmacology, and clinical manifestations and did not verify the sequencing results directly. In the future, T cells can be separated for cell culture, CFDT-containing serum can be used for intervention, and potential target gene detection can be performed on different cell subsets.

## 5 Conclusion

To summarize, this study combines scRNA-seq, network pharmacology, and clinical observation for the first time. It innovatively explores the effects of traditional Chinese medicine on obese PCOS from an immune perspective. Bioinformatics analysis and clinical observation results depicted that patients with PCOS, particularly obese patients with PCOS, have changes in the immune microenvironment. Quercetin, carvacrol, β-sitosterol, cholesterol, and nobiletin are the main active ingredients of CFDT. These ingredients may improve the immune microenvironment of obese patients with PCOS through targets such as TP53, AKT1, STAT3, JUN, and SRC and are based on pathways such as TRC signaling pathways, thereby improving clinical outcomes. These findings are expected to guide the application and further development of CFDT for treating PCOS.

## Data Availability

The data on human single-cell RNA sequencing are not readily available because of patient privacy or ethical restrictions. Reasonable requests to access the datasets should be directed to corresponding author.
